# Sustainable Substitution of Petroleum-Based Processing Oils with Soybean-Derived Alternatives in Styrene–Butadiene Rubber: Effects on Processing Behavior and Mechanical Properties

**DOI:** 10.3390/polym17152129

**Published:** 2025-08-01

**Authors:** Yang-Wei Lin, Tsung-Yi Chen, Chen-Yu Chueh, Yi-Ting Chen, Tsunghsueh Wu, Hsi-Ming Hsieh

**Affiliations:** 1 Department of Chemistry, National Changhua University of Education, Changhua City 50007, Taiwan; m1325007@mail.ncue.edu.tw (C.-Y.C.); m1225010@mail.ncue.edu.tw (Y.-T.C.); 2 Innova Rubber Co., Ltd., Changhua City 50067, Taiwan; innova.qa.st2@innovatires.com.tw; 3 Department of Chemistry, University of Wisconsin-Platteville, Platteville, WI 53818-3099, USA; wut@uwplatt.edu

**Keywords:** styrene–butadiene rubber (SBR), soybean oil (SBO), epoxidized soybean oil (ESBO), green and sustainable materials, mechanical properties, petroleum oil replacement

## Abstract

This study evaluates the replacement of petroleum-based naphthenic oil with four types of soybean-derived alternatives—virgin soybean oil (SBO), epoxidized SBO (ESBO), expired SBO, and recycled SBO—in styrene–butadiene rubber (SBR) composites. The materials were tested in both staining rubber (SR) and non-staining rubber (NSR) systems to assess processing characteristics, mechanical performance, and environmental durability. Among the alternatives, SBO demonstrated the best overall performance, improving processability and tensile strength by over 10%, while ESBO enhanced ozone resistance by 35% due to its epoxide functionality. Expired and recycled SBOs maintained essential mechanical properties within 90% of virgin SBO values. The full replacement of CH450 with SBO in tire prototypes resulted in burst strength exceeding 1000 kPa and stable appearance after 5000 km of road testing. To validate industrial relevance, the developed green tire was exhibited at the 2025 Taipei International Cycle Show, attracting interest from international buyers and stakeholders for its eco-friendly composition and carbon footprint reduction potential, thereby demonstrating both technical feasibility and commercial viability.

## 1. Introduction

Recent studies highlight the growing imperative to develop environmentally sustainable tires, driven by both industrial innovation and environmental concerns [1]. One study emphasizes the integration of renewable materials, such as soybean oil (SBO), rice husk silica, and dandelion-derived rubber, into tire formulations to reduce dependency on fossil-based inputs and cut carbon emissions [1,2,3]. Manufacturers are also advancing chemical recycling technologies, such as pyrolysis, to recover valuable resources like carbon black and synthetic oils, helping to reduce the environmental impact of end-of-life tires. Despite these advancements, tire and road wear particles (TRWPs) remain a significant unresolved issue, contributing to microplastic pollution and potentially toxic chemical release [1]. Although the tire industry is actively exploring ways to address this, further research is required to fully understand and mitigate these effects. Together, these findings underscore that while substantial progress is being made toward greener tire production, achieving a truly sustainable tire also requires tackling broader lifecycle challenges, including raw material sourcing, processing efficiency, and end-of-life waste management. Among various natural oils, virgin SBO was selected in this study due to its favorable chemical characteristics and compatibility with rubber matrices. SBO consists predominantly of triglycerides with unsaturated long-chain fatty acids, averaging ~4.6 double bonds per molecule. This high unsaturation facilitates reactivity during vulcanization, allowing SBO to act as a reactive plasticizer that improves filler dispersion and reduces viscosity. Its solubility parameter (~16.6–16.7 MPa^1^/^2^) aligns well with common rubbers, enhancing miscibility and internal lubrication. Compared to other natural oils, SBO offers advantages in cost, availability, and chemical compatibility, making it a practical and effective bio-based alternative to petroleum-based plasticizers.

For example, Zhu et al. developed a bio-based silane coupling agent (SO-Si) from SBO via a solvent-free thiol–ene click reaction for green tire tread applications [4]. Acting as both a plasticizer and coupling agent, SO-Si significantly improved rubber–silica interaction, enhancing wear resistance (by 30%) and skid resistance (29%) and reducing heat generation (26%) and rolling resistance (50%) compared to conventional systems. However, high grafting levels led to gelation, limiting processing, and UV-initiated synthesis requires careful control for scalability. Despite these challenges, SO-Si presents a promising route for sustainable, high-performance rubber composites. Another study developed bio-based plasticizers by reacting SBO with sulfur to tailor double bond content [5]. Modified soybean oil (MSO) is synthesized from SBO and sulfur, aiming to reduce the double bond quantity of SBO and avoid harmful effects on the crosslink density and mechanical properties of rubber. MBO-6% showed the best balance of properties, improving processability, mechanical strength, wear resistance, and aging stability while reducing curing time. The solvent-free synthesis and use of renewable resources support sustainable material development. However, excessive sulfur content (e.g., MSO−9%) impairs mechanical performance, underscoring the need for precise control. Further studies are needed to assess long-term stability and compatibility with other rubber types [6]. The literature examines vegetable oils such as palm, soybean, and linseed oils as eco-friendly alternatives to petroleum-based processing aids in rubber composites. These oils enhance filler dispersion and reduce rolling resistance while offering renewability and low toxicity. However, high loadings may hinder vulcanization, and most studies focus on carbon black-filled systems, with limited data on silica and CaCO_3_. Further research is needed to optimize performance and evaluate industrial scalability. Furthermore, existing studies are predominantly confined to the laboratory scale, with an emphasis on investigating the interactions between chemical components [3,7,8]. To date, there is a lack of practical application data, particularly concerning the manufacturing of bicycle tires and the evaluation of their wear performance and stability under actual operating conditions.

In response to global climate change and the depletion of non-renewable resources, this study explores a sustainable alternative to conventional petroleum-based processing oils used in bicycle tire manufacturing. Petroleum-derived oils are linked to high carbon emissions, as well as long-term environmental and health concerns. This study introduces an innovative green strategy by substituting petrochemical oils with plant-based alternatives, specifically SBO, epoxidized SBO (ESBO), recycled SBO (r-SBO), and expired SBO (e-SBO), for rubber outer tire formulations. These bio-based oils are renewable and biodegradable and align with the principles of carbon neutrality and circular economic development [9,10]. This research contributes to Taiwan’s green manufacturing transformation and provides a replicable model for sustainable industrial upgrading in the global rubber and tire sectors. This study demonstrates a viable pathway for industrial decarbonization by incorporating SBO derivatives, including expired and recycled variants, as substitutes for fossil-derived oils. Furthermore, partnerships with local edible oil suppliers facilitate the establishment of a reusable waste oil recycling system, which improves material circularity and resource efficiency [11]. This closed-loop model not only reduces waste but also enhances local economic resilience.

With the increasing demand for sustainable bicycle products in markets such as Europe and North America, green tires have become a competitive advantage. By utilizing domestically sourced SBO, this study achieves excellent processing behavior, mechanical durability, and thermal aging resistance, thereby enhancing both product performance and Taiwan’s position in the global green tire industry. This study aims to create innovative green rubber formulations using non-edible vegetable oils as exciting alternatives to traditional petroleum-based processing oils. Our goals include the following: (1) a thorough comparison of the physicochemical properties of various vegetable oils, such as SBO and ESBO, with traditional petroleum-derived oils; (2) a deep dive into the benefits and challenges of using SBO and ESBO in rubber compounding; (3) establishing strong technical specifications for these vegetable oils as rubber plasticizers; (4) rigorously evaluating their effects on rubber processing, mechanical performance, and long-term durability; and (5) pinpointing the optimal usage ratios for achieving outstanding products. Additionally, we will conduct comprehensive testing of green tires to ensure they are structurally sound and reliable for the long haul. Our mission is to replace petroleum-based oils with renewable, biodegradable, and carbon-reducing vegetable oils, resulting in eco-friendly bicycle tires that not only meet but exceed industry performance, durability, and safety standards [12]. By championing carbon neutrality and supporting circular economy initiatives, we are paving the way for a brighter, greener future in transportation.

## 2. Materials and Methods

### 2.1. Materials

The materials used in this study were sourced from commercial suppliers and applied without further purification. Butadiene rubber (BR0150) and styrene–butadiene rubber (SBR1502) were both obtained from TSRC Corporation (Kaohsiung, Taiwan). Ethylene–propylene–diene monomer (EPDM) rubber was supplied by DuPont Taiwan Ltd. (Taipei, Taiwan). Petroleum-based naphthenic oil (CH450), used as a conventional processing aid, was acquired from ChouFeng Enterprise Co., Ltd. (New Taipei City, Taiwan). SBO, ESBO, e-SBO, and r-SBO were supplied by Taisun Enterprise Co., Ltd. (Changhua, Taiwan). Carbon black was provided by International CSRC Investment Holdings Co., Ltd. (Taipei, Taiwan), while silica (white carbon black) was purchased from Evermore Group (Taipei, Taiwan). Ultrafine calcium carbonate was sourced from YunCheng Chemical Industry Co., Ltd. (Tainan, Taiwan). Zinc oxide was obtained from Diamonchem International Co., Ltd. (Changhua, Taiwan), and a silica activator was purchased from Evergreen Chemical Industrial Co., Ltd. (Taoyuan, Taiwan). Sulfur was provided by Eastech Chemical Co., Ltd. (Changhua, Taiwan). Vulcanization accelerators and a pre-vulcanization inhibitor were all obtained from Willingchem New Materials Technology Co., Ltd. (Puyang, China).

### 2.2. Preparation of Rubbers

Rubber compounds were prepared using a two-stage mixing process. According to the designed formulation, 40% rubber, 35% reinforcing fillers (e.g., carbon black, silica), 5% plasticizers (e.g., petroleum-based naphthenic oil, SBO, and ESBO), 12% antioxidants, and 3% other base additives were weighed in specific proportions and loaded into an internal mixer (or two-roll open mill) for the first mixing stage. This study will examine the composition of processing oil with varying quantities in 5% plasticizer, as illustrated in Table 1.

During this stage, ingredients were mixed at a controlled temperature and rotor speed to ensure uniform dispersion within the rubber matrix. After the completion of the first mixing stage, the compound was discharged and sheeted out, followed by a cooling process to prevent the premature reaction of heat-sensitive additives. Once the compound temperature dropped to an appropriate level (cool down to room temperature), the second mixing stage was conducted. In this stage, curing agents, including sulfur and accelerators, were added to the base compound using the open mill, facilitating uniform distribution while minimizing premature crosslinking. The fully mixed compound was then molded into standard test specimens through compression molding at 165 °C under a pressure of 5 MPa for 6 min to achieve complete vulcanization. The cured sheets were subsequently conditioned and used for various physical property evaluations according to relevant testing standards.

**Figure 1:**
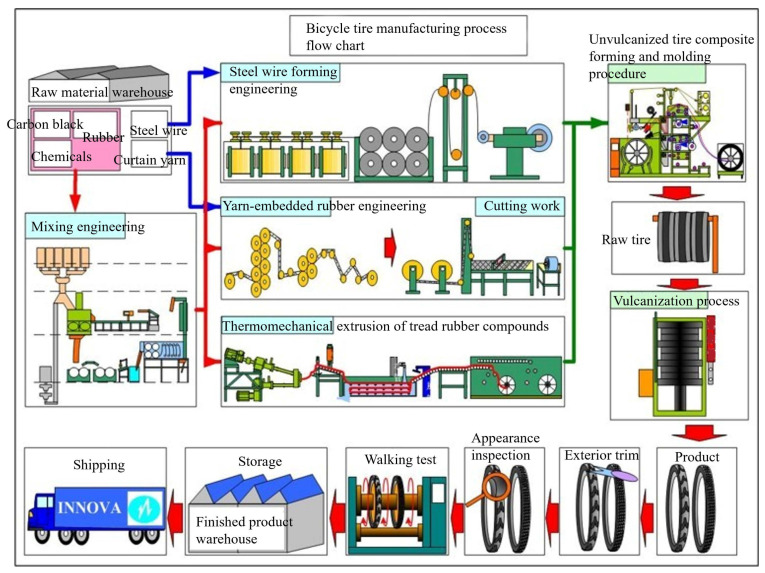
A flow chart of the bicycle tire manufacturing process.

The preparation of the bicycle tire followed a multi-stage manufacturing process, as shown in Figure 1. Initially, raw materials, including rubber, additives, steel wires, and reinforcing fabrics, were weighed according to the formulation design (rubber: 60%; steel wires: 8%; reinforcing fabrics: 32%). These ingredients were introduced into a kneading process (internal or open mill mixing) to prepare rubber compounds with the desired processing and mechanical properties. After mixing, the rubber compound was processed into various semi-finished components such as tread strips, sidewall strips, inner liners, bead wires, and breaker fabrics. These components were fabricated via extrusion, calendaring, and cutting operations according to design specifications. Subsequently, all components were assembled in a tire-building machine following a specific layering sequence to form the bicycle tire (an uncured tire prototype). The bicycle tire was then subjected to vulcanization in a curing press at 165 °C under a pressure of 15 MPa for 5 min. This crosslinking process transformed the raw rubber matrix into a dimensionally stable and durable final product with the mechanical properties required for bicycle tire applications.

### 2.3. Characterization

The mechanical and physicochemical properties of the rubber composites were characterized using a series of standardized instruments. Tensile strength and elongation at break were measured using a universal tensile testing machine (Model TS-2000) manufactured by EKTRON TEK Co., Ltd. (Changhua County, Taiwan). To assess the curing behavior, a sealed-type rotor-less rheometer (Model EKT-2000S, EKTRON TEK Co., Ltd.) was employed, enabling the precise determination of parameters such as scorch time and optimum cure time. The processability of the compounds was further evaluated using a Mooney viscometer (Model EKT-2001M, EKTRON TEK Co., Ltd., Changhua County, Taiwan). Abrasion resistance testing was conducted using a DIN-type abrasion tester (Model PT-3010) provided by PERFECT INTERNATIONAL INSTRUMENTS Co., Ltd., based in Taichung City, Taiwan. Thermal aging performance was evaluated using a fully automatic ventilated aging oven (Model GT-7017-L, GOTECH TESTING MACHINES INC., Taichung, Taiwan), while ozone resistance was assessed by means of an ozone chamber (Model SUGA OMS-L) supplied by SANPANY INSTRUMENTS Co., Ltd., Taipei, Taiwan. All measurements were performed under controlled laboratory conditions to ensure data reliability and reproducibility for subsequent analysis.

## 3. Results and Discussion

### 3.1. Properties of Soybean Oil

To evaluate the feasibility of using edible oil products as eco-friendly plasticizers in bicycle tire rubber compounds, the physicochemical properties of four non-petroleum-based vegetable oils, namely SBO, r-SBO, e-SBO, and ESBO, were first characterized. As shown in Table 2, the viscosities of SBO, r-SBO, and e-SBO at 40 °C were comparable to that of CH450, indicating suitable flowability for rubber compounding. In contrast, ESBO exhibited a significantly higher viscosity (212.9 mm^2^/s), suggesting lower flowability. This high viscosity is anticipated to enhance mechanical strength and elasticity in the rubber matrix [13]. Specific gravity data showed that ESBO possessed the highest value (0.979), attributable to its high molecular polarity, while e-SBO had the lowest (0.933), implying varying degrees of lubrication potential [14]. Initial boiling point, freezing point, and flash point analyses confirmed that all oils possess acceptable storage safety. Spectral analysis revealed distinctive epoxy functional group absorption peaks for ESBO, indicating successful chemical modification. UV–vis analysis further suggested a reduction in double bond content, lowering oxidative susceptibility. Overall, SBO emerges as the most suitable base replacement due to its stability and biodegradability, whereas r-SBO and e-SBO offer cost and sustainability advantages but pose challenges in consistency. ESBO holds potential for performance enhancement but faces processing and cost constraints.

At present, the evaluation of oil products based solely on their physicochemical properties does not allow for a complete prediction of their actual impact on rubber compound performance and finished product quality. Therefore, this study proposes a practical investigation by substituting petroleum-based processing oils with four types of vegetable oils (SBO, r-SBO, e-SBO, and ESBO). In the first phase, the systematic replacement of petroleum oil with SBO and ESBO at the 25 wt%, 50 wt%, 75 wt%, and 100 wt% levels is conducted to assess its effects on the processing behavior, mechanical strength, aging resistance, and durability of rubber compounds. Based on the resulting data, a comprehensive evaluation is then carried out to determine the suitability, limitations, and optimization strategies for each oil type in sustainable rubber formulation development. This approach addresses both material property gaps and practical performance, offering insights for future bio-based processing oil applications in green rubber technologies.

### 3.2. Characterization of Staining Rubbers with Soybean-Derived Alternatives

**Figure 2:**
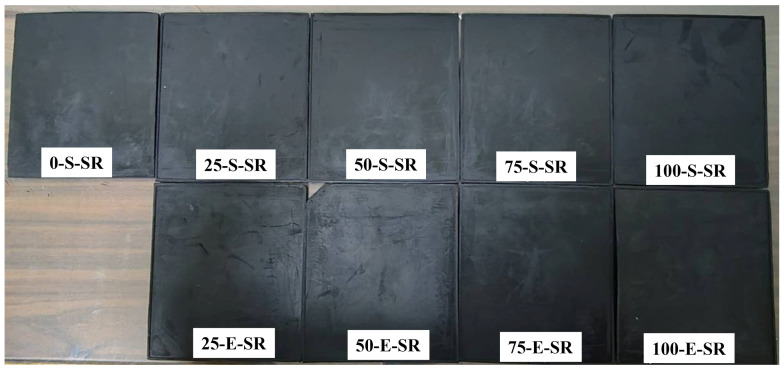
Photographic images of the SRs with different weight percentages of soybean-derived alternatives.

According to Table 1, staining rubber (SR) formulations were modified by substituting petroleum-based oil with SBO and ESBO at varying proportions. As illustrated in Figure 2, there were no visually discernible differences in the surface color, texture, or integrity of the rubber specimens across all formulations. This indicates that the type and ratio of bio-based oil used had minimal impact on the macroscopic appearance of the final rubber products. This result suggests that, despite variations in viscosity and polarity between SBO and ESBO, these factors do not significantly influence the rubber’s esthetic or surface morphology under the tested processing conditions. This finding supports the practical feasibility of incorporating bio-based oils in rubber compounding without compromising visual uniformity, a key factor for end-use applications in consumer-facing products [15,16].

**Figure 3:**
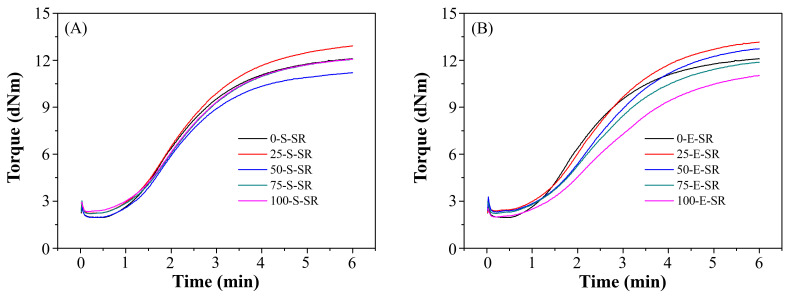
Rheographs of the SRs with different weight percentages of (**A**) S and (**B**) E soybean-derived alternatives.

To evaluate processability, rubber compounds incorporating various levels of SBO and ESBO were analyzed using Mooney viscosity (MV) and rheographs (Figure 3), including TS1 (scorch time) and TC90 (optimum cure time). As shown in Table 3, MV and rheological responses showed limited variation across different SBO replacement levels (X = 25, 50, 75, 100). However, compounds in the X-E-SR group (using ESBO) exhibited consistently higher MV values, attributed to the significantly higher viscosity of ESBO, which hinders flow and mixing efficiency [17]. TS1 values remained relatively stable across all oil ratios, indicating a similar onset of vulcanization. In contrast, TC90 increased with higher ESBO content, suggesting prolonged cure time. This is likely due to the difficulty in dispersing ESBO uniformly in the rubber matrix, which delays crosslinking [18]. Overall, ESBO’s high viscosity negatively impacts both fluidity and curing efficiency, while SBO maintains favorable processing characteristics.

The interaction between processing oils and rubber compounds is fundamental to both processing behavior and the final performance of vulcanizates. SBO, a triglyceride-based bio-oil rich in unsaturated fatty acids, exhibits a solubility parameter of approximately 16.2–16.7 MPa^1^/^2^. This value closely matches that of common diene rubbers such as natural rubber, polybutadiene rubber, and styrene–butadiene rubber, indicating favorable miscibility and internal plasticization behavior. As a result, the addition of SBO facilitates polymer chain mobility, lowers Mooney viscosity, and improves the dispersion of fillers during compounding.

However, the high degree of unsaturation in SBO introduces challenges during vulcanization. The double bonds in the fatty acid chains are reactive and may compete with the rubber matrix for the vulcanizing agent (e.g., sulfur), potentially leading to a reduction in crosslink density. This effect was reflected in our results, where SBO-plasticized compounds exhibited a slightly reduced 300% modulus and hardness compared to those using conventional aromatic oil, although tensile strength and elongation at break remained acceptable. This trade-off highlights the dual role of virgin SBO as both a plasticizer and a reactive species within the curing system.

These findings are consistent with previous studies reporting that while virgin vegetable oils enhance processing characteristics and offer environmental advantages, their high reactivity can influence vulcanization kinetics and mechanical properties if not properly balanced with the formulation strategy. Future work may explore oil modification or synergistic use with co-additives to optimize performance further.

Table 4 presents the influence of various oil replacement ratios on the physical properties of SR. Hardness and specific gravity values remained within acceptable ranges, showing minimal variation across formulations. However, noticeable differences were observed in tensile and tear strengths. In the X-E-SR group, tensile strength was lower than that in the X-S-SR group, likely due to the higher viscosity of ESBO impeding uniform raw material dispersion, which negatively affected crosslinking efficiency and mechanical integrity [18]. Additionally, the inherent formulation characteristics of SR, compared with non-staining rubber (NSR), may contribute to significant variation in tear strength, with SR consistently exhibiting higher tear resistance. This enhanced tear strength is advantageous for applications requiring extended durability and resistance to mechanical wear, such as tire treads and industrial rubber components [19].

After exposing the X-Y-SR samples (X = 25, 50, 75, 100; Y = S, E) to an ozone-rich environment (50 ppm) for 12 h, surface cracking was examined to evaluate ozone resistance. The results, summarized in Table 5, show no significant difference in ozone-induced degradation among samples using CH450, SBO, or ESBO as processing oils. This indicates that replacing CH450 with vegetable-derived oils does not compromise the rubber’s ozone resistance. Interestingly, the 100-E-SR formulation exhibited marginally enhanced ozone resistance. This improvement is likely due to the reduced content of carbon–carbon double bonds (C = C) in ESBO, resulting from the epoxidation process. As C = C bonds are primary targets for ozone attack, their reduction decreases the vulnerability of the rubber matrix to oxidative cleavage [20]. Consequently, ESBO contributes to enhanced oxidative stability and reduced surface cracking under ozone exposure, supporting its viability as a sustainable alternative to petroleum-based plasticizers in rubber formulations.

Table 5 presents the AKRON abrasion results for X-Y-SR formulations (X = 25, 50, 75, 100; Y = S, E), which were used to assess the wear resistance of the rubber compounds. The data indicate that the wear loss values of the X-S-SR group were comparable to those of the control group using CH450 petroleum-based processing oil (0-S-SR). This suggests that substituting CH450 with SBO does not significantly impair the wear resistance of the rubber. Conversely, the ESBO-containing samples did not exhibit the anticipated improvement in wear resistance. A plausible explanation for this outcome is the high viscosity of ESBO, which may hinder its uniform dispersion during compounding [18]. This insufficient dispersion could lead to uneven filler distribution and suboptimal crosslinking, resulting in a less compact rubber matrix. Such microstructural looseness may, in turn, reduce the compound’s resistance to mechanical abrasion. These findings highlight the importance of balancing oil viscosity and compatibility to achieve optimal reinforcement in rubber formulations.

Number of cracks

N: No cracks.
A: A few cracks (less than 20).
B: Many cracks (assuming that the number of cracks can be determined).
C: An infinite number of cracks (when unable to calculate the number of cracks).
E: The edge of the test piece.
e: Cracks on the edge of the test piece.

Crack size and depth

1. Those that cannot be seen with the naked eye but can be seen with a 10x magnifying glass.
2. Cracks that can be seen with the naked eye.
3. Crack growth, size less than 1 mm.
4. Crack growth, size more than 1 mm and less than 3 mm.
5. Cracks that grow larger than 3 mm or may cause fracture.

The physical properties of the X-Y-SR samples (X = 25, 50, 75, 100; Y = S, E) were evaluated before and after thermal aging at 100 °C for 72 h to assess the aging durability of each experimental group (as summarized in Table 6). In general, key mechanical properties, such as tensile strength and elongation at break, declined to varying extents across all X-Y-SR groups following aging. Notably, within the same processing oil system, the 100-Y-SR (Y = S, E) formulations exhibited comparatively lower degradation rates, suggesting enhanced thermal aging stability. This improved performance may be attributed to the homogeneous oil composition at full replacement, which promotes consistent dispersion and compatibility within the rubber matrix. In contrast, formulations with mixed replacement ratios (X = 25, 50, 75) may suffer from inconsistencies in oil polarity, viscosity, or reactivity, potentially leading to non-uniform dispersion and irregular crosslinking during processing. These microstructural inconsistencies could compromise network integrity and contribute to the more pronounced deterioration in mechanical properties observed after aging.

### 3.3. Characterization of Non-Staining Rubbers with Soybean-Derived Alternatives

**Figure 4:**
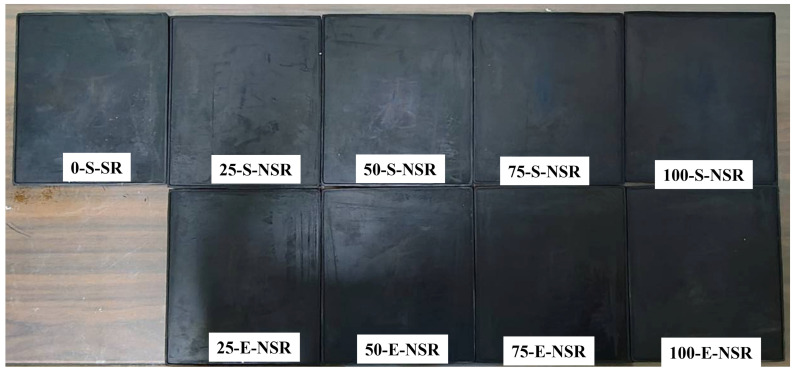
Photographic images of the NSRs with different weight percentages of soybean-derived alternatives.

To evaluate the visual effect of vegetable oil substitution in NSR, samples were prepared using different replacement ratios (X = 0, 25, 50, 75, 100) and oil types (S and E), coded as X-Y-NSR. As shown in Figure 4, the appearance of the rubber samples across all formulations exhibited no significant visual differences compared to the CH450-based control. This suggests that substituting CH450 with either SBO or ESBO, even at full replacement levels, does not adversely affect the surface uniformity or overall visual characteristics of NSR compounds. This observation aligns with the findings for SR, indicating the good compatibility of bio-based oils with both rubber types.

**Figure 5:**
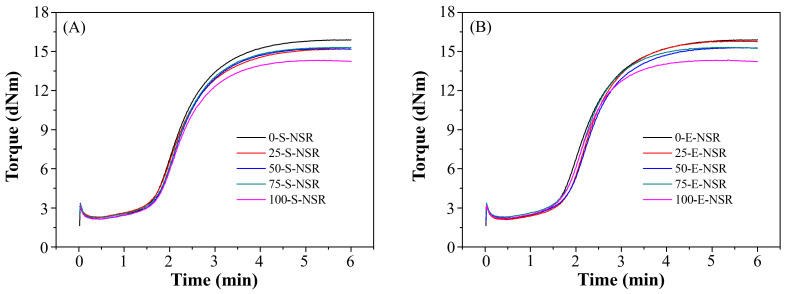
Rheographs of the NSRs with different weight percentages of (**A**) S and (**B**) E soybean-derived alternatives.

The processing properties of NSR compounds were evaluated by replacing CH450 naphthenic oil with SBO and ESBO in varying proportions. MV values and rheological parameters, including TS1 and TC90, were measured from rheographs (Figure 5), and the results are summarized in Table 7. Across all experimental groups (X-Y-NSR), MV values were slightly higher than the standard range (53–63). However, within each oil type group, variations in the replacement ratio had minimal impact on MV, suggesting that the oil substitution ratio does not significantly affect viscosity and that mixing stability remains acceptable. TS1 values remained within a reasonable range and exhibited no clear trend with changes in oil content, indicating comparable initial vulcanization reactivity for both SBO and ESBO. Notably, the TC90 values in the SBO group remained relatively stable across ratios, whereas the ESBO group showed a slight decrease in TC90 with an increasing replacement ratio, potentially reflecting enhanced cure kinetics due to the functional characteristics of ESBO.

The physical properties of the X-Y-NSR compounds are summarized in Table 8. Replacing CH450 with vegetable oils revealed a mixed impact on material performance, with specific properties improving while others declined. Specifically, hardness and specific gravity remained relatively unchanged across all X-Y-NSR groups (X = 25, 50, 75, 100; Y = S, E), suggesting that the substitution had a limited effect on the density and mass of the rubber matrix. Within each oil type, changes in the replacement ratio did not lead to significant fluctuations in these physical parameters, indicating good formulation consistency. However, for all X-Y-NSR groups (Y = S, E), a reduction was observed in ductility-related mechanical properties, including tensile strength, elongation at break, and tear strength. These decreases suggest that while vegetable oil substitution is feasible, it may adversely affect the elasticity and toughness of NSR to some extent, warranting further optimization in formulation or processing conditions.

As presented in Table 9, NSR compounds demonstrated excellent ozone resistance after substituting CH450 with varying proportions of SBO and ESBO. Across all replacement levels (25%, 50%, 75%, and 100%), the NSR samples exhibited no observable surface cracking after exposure to an accelerated aging environment of 50 ppm ozone for 12 h. These results suggest that incorporating vegetable oils, whether SBO or ESBO, does not compromise the ozone durability of the NSR formulation. The absence of degradation indicates that such bio-based oils are viable alternatives to petroleum-based processing oils in applications requiring ozone stability.

The AKRON wear test results for X-Y-NSR samples (X = 0, 25, 50, 75, 100; Y = S, E), presented in Table 9, reveal a trend consistent with that observed in the SR formulations. Specifically, the X-Y-NSR groups (Y = E) exhibit comparatively higher wear loss. This suggests that when ESBO is used to replace CH450, even in NSR systems, its high intrinsic viscosity continues to hinder efficient dispersion during mixing. Poor dispersion likely leads to inhomogeneous filler distribution and suboptimal crosslinking, resulting in a looser rubber matrix and diminished abrasion resistance. In contrast, the wear loss in SBO-based NSR formulations remains close to that of the reference sample (X = 0), indicating more favorable processing characteristics and better overall wear performance.

The results of the heat aging test conducted at 100 °C for 72 h indicate that the physical properties of the X-Y-NSR formulations declined only slightly after aging (as summarized in Table 10). There were minimal variations between the replacement ratios within the same oil type, suggesting stable thermal performance. The 100-S-NSR formulation demonstrated a strong retention of mechanical properties, likely due to effective oil dispersion and consistent crosslinking during processing. These findings suggest that NSR formulations, particularly those with the complete substitution of SBO, exhibit good resistance to thermal aging and are suitable for applications that require heat stability.

### 3.4. Effects on Processing Behavior and Mechanical Properties Using e-SBO and r-SBO

**Figure 6:**
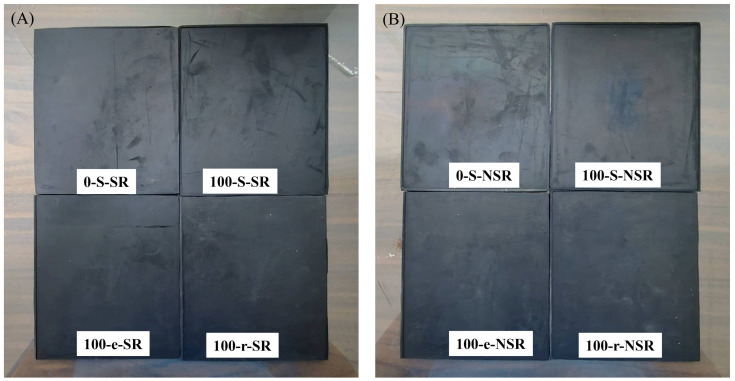
Photographic images of the (**A**) SRs and (**B**) NSRs with soybean-derived alternatives.

To promote environmental sustainability and enhance resource recycling, this study investigates the feasibility of replacing CH450 naphthenic oil with r-SBO and e-SBO. The formulation codes for the oil-replaced rubber compounds are provided in Table 1. As shown in Figure 6, regardless of the rubber type (SR or NSR), the appearance of samples made with 100% r-SBO and 100% e-SBO shows no significant difference compared to those prepared with CH450. This indicates that the substitution does not negatively affect the visual quality of the final rubber products.

**Figure 7:**
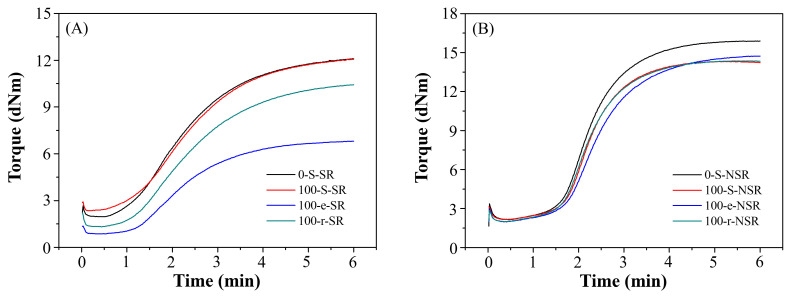
Rheographs of the (**A**) SRs and (**B**) NSRs with 100% fresh, expired, and recycled soybean-derived alternatives.

Table 11 displays the processing performance of 100-Y-SR and 100-Y-NSR (where Y = e, r). The measured MV values for 100-e-SR and 100-r-SR were 38.18 and 37.41, respectively, significantly below the standard processing range (SR: 56–66; NSR: 53–63). This notable reduction in MV values can likely be attributed to changes in the chemical composition and oxidation levels of e-SBO or r-SBO. Specifically, oxidation or degradation during storage or prior use may have led to lower-molecular-weight components or increased polarity. These factors can lead to strong plasticizer behavior in the rubber matrix, altering the compound’s viscosity and causing the rubber to become excessively soft during processing. The rheological parameters, including TS1 and TC90, were measured from the rheographs (Figure 7).

The results of the physical property tests for the use of 100-Y-SR and 100-Y-NSR (where Y = e, r) show that the fundamental characteristics of these substitute oils are similar to those of SBO. As a result, the differences in mechanical performance are minimal. Parameters such as hardness, tensile strength, elongation at break, M300, and tear strength do not significantly differ from those observed in the 100-S-SR and 100-S-NSR groups, as summarized in Table 12.

Based on the data presented in Table 13, both 100% e-SBO and 100% r-SBO rubber compounds (SR, NSR) show ozone and abrasion resistance similar to that of the reference group using CH450. In the ozone resistance test, the NSR formulations containing 100% e-SBO or r-SBO exhibited no surface cracking, performing comparably to the X-S-NSR (where X = 0, 100) group. Additionally, the AKRON wear test results indicate that the wear losses of 100-e-SR and 100-r-SR are consistent with those of the formulations based on 100-S-SR. This suggests that substituting CH450 with 100% e-SBO or r-SBO does not negatively affect wear resistance. These findings highlight the potential for using waste-based SBO as sustainable processing oil alternatives without compromising key performance attributes.

As shown in Table 14, after thermal aging at 100 °C for 72 h, the physical properties of rubber compounds made with 100% e-SBO (100-e-SR) and 100% r-SBO (100-r-SR) showed a significant decline, particularly in tensile strength, elongation, and modulus. This decline suggests that oxidative degradation and increased acidification in expired and recycled SBO may disrupt the rubber matrix and impair the stability of the crosslinks, leading to reduced resistance to thermal aging. In contrast, the physical properties of the NSR formulations using the same oils remained relatively stable after aging. This stability indicates that the NSR system has a greater tolerance to variations in oil quality, likely due to enhanced structural integrity and improved filler–matrix interaction. These factors help maintain crosslink density and mechanical performance under thermal stress.

### 3.5. Evaluation Performance of Tire Fabricated with 100-S-SR

The test results indicate that 100% SBO outperformed other oil types in terms of processability, the retention of physical properties, and resistance to thermal aging. These benefits position SBO as a promising eco-friendly alternative to conventional mineral oils used in rubber compounding. As a result, 100% SBO was chosen as the preferred sustainable processing oil for tire formulations. Additionally, the large-scale production of NSR compounds using 100% SBO demonstrated consistent processing behavior and mechanical performance, remaining within the acceptable specification ranges required for commercial applications. It is noteworthy that the vulcanization time applied in the preparation of laboratory test sheets differs from that used in actual tire manufacturing. This discrepancy arises from the fundamental differences in material structure and processing configuration. In the small-scale laboratory tests, vulcanization was conducted on rubber-only specimens, enabling a focused evaluation of the compound’s curing behavior. In contrast, the tire vulcanization process involves complex, multi-layered assemblies incorporating not only rubber but also reinforcing materials such as steel belts and textile plies. These additional components significantly affect heat conduction and the overall curing dynamics. Consequently, the industrial vulcanization process requires an extended curing time and temperature adjustments to accommodate the thicker tread rubber and ensure consistent crosslinking throughout the tire structure. Given that this study originates from an industry–academia collaborative project targeting practical application, the vulcanization conditions for tire production were optimized accordingly to reflect real-world manufacturing constraints and performance criteria.

**Figure 8:**
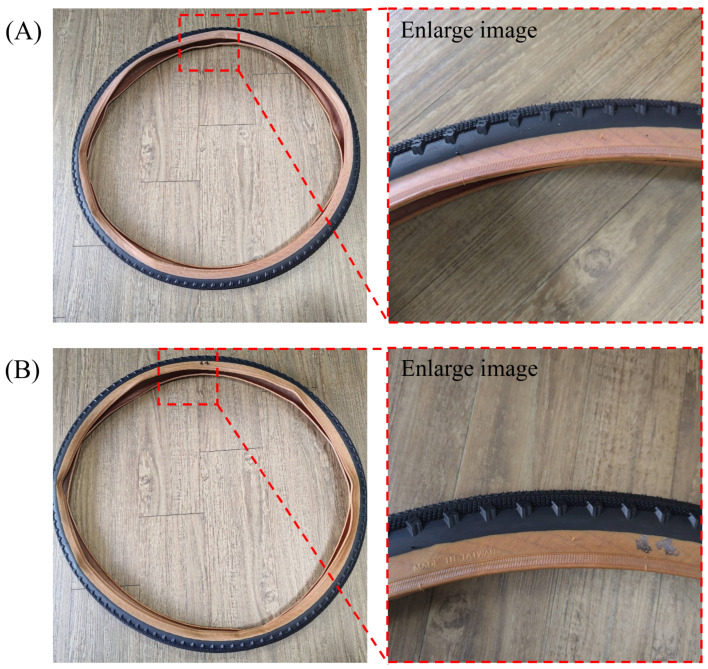
Photographic images of (**A**) the tire and (**B**) the exposed tire that was produced with 100-S-SR. The Chinese meaning is “exposure” and is used as a record mark for experiments.

Figure 8A demonstrates that a tire manufactured with 100% SBO as the processing oil exhibits outstanding appearance quality and environmental stability. Remarkably, there were no visible defects, contamination, or phase separation among the rubber components. After seven days of direct sunlight exposure, the tire’s surface remained flawless, showing no signs of discoloration, exudation, or deposits, which underscores its exceptional weather resistance, as shown in Figure 8B. These compelling results indicate that SBO is compatible with the rubber matrix and effectively preserves the esthetic and structural integrity of the tire when faced with environmental challenges. This aligns seamlessly with prior research demonstrating that SBO-based rubbers enhance dispersion, elevate flexibility, and deliver impressive aging resistance in tire applications [2,3].

The burst test results highlight the remarkable performance of the outer tire created with the SBO-based rubber formulation. It endured a water pressure of 1000 kPa for 2 min without any sign of failure. There were no visible cracks, leaks, or deformations, which strongly attests to its outstanding structural integrity and internal pressure resilience. This impressive performance demonstrates that the rubber matrix and compounding strategy employed are practical and capable of maintaining dimensional stability and mechanical strength under extreme pressure conditions [4,5]. Such attributes meet the essential safety and durability standards for bicycle tires, reinforcing their reliability during high-pressure situations in real-world applications.

Table 15 demonstrates that tires made with 100% SBO as a processing oil provide exceptional durability in real-world conditions. After 5000 km of continuous driving at 40 km/h, no visible damage, such as cracks, delamination, or surface degradation, was observed. This impressive performance highlights the tires’ outstanding wear resistance, rubber cohesion, and oxidative stability, all critical factors in determining tire reliability. These results confirmed that SBO improves rubber flexibility and cold-weather traction and enhances overall compound performance while minimizing environmental impact. Thus, SBO is more than just an alternative; it is a vital component that meets the rigorous long-term performance standards for sustainable, high-durability tires [2,6].

**Figure 9:**
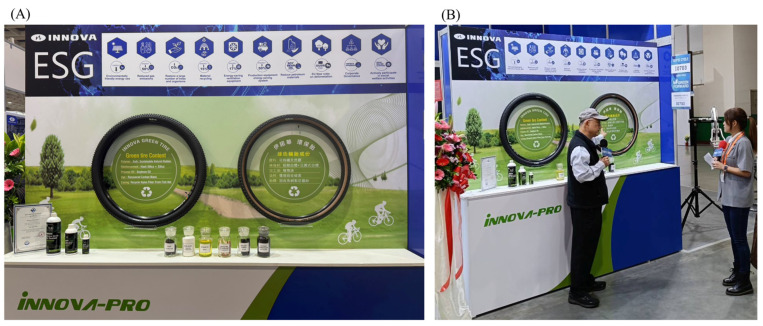
(**A**) The exhibition of the eco-friendly bicycle tire prototype at TAIPEI CYCLE 2025. (**B**) Mr. Hsi-Ming Hsieh, a corresponding author of this study, was interviewed by media representatives during the exhibition, highlighting the scientific and industrial relevance of the presented results.

As shown in Figure 9, the innovative bicycle tire developed in this study was officially exhibited at the 2025 Taipei International Cycle Show (TAIPEI CYCLE 2025), one of the most prominent and influential exhibitions in Asia’s bicycle industry. During the exhibition, the product attracted significant attention from international media and industry stakeholders due to its eco-friendly materials, high-performance characteristics, and potential for carbon footprint reduction. Numerous buyers and companies inquired about future development plans and the possibility of procurement or licensing agreements. This public presentation not only validated the technical feasibility of the research outcomes but also demonstrated the product’s market readiness and strong potential for industrial application. The visibility and recognition at such a reputable platform underscore the value and impact of the developed green tire technology.

### 3.6. Comparative Analysis of Bio-Based and Petroleum-Based Processing Oils in Rubber Composites

The global shift toward sustainability has intensified efforts to replace petroleum-derived plasticizers with bio-based oils in rubber compounding. Our findings, consistent with previous reports [4,5,6,7,8,18], confirm that virgin SBO enhances compound processability and mechanical properties.

In the processing characteristics field, virgin SBO effectively lowers Mooney viscosity, facilitating better mixing and processing, aligned with the results from Xu et al. [5] and Cataldo et al. [8]. In contrast, ESBO increases viscosity and prolongs cure time due to its high kinematic viscosity [18]. e-SBO and r-SBO exhibited even lower viscosities, resulting in overly soft compounds, likely due to degradation-induced molecular breakdown. For mechanical strength, SBO-plasticized compounds showed a >10% improvement in tensile strength, though with a slightly reduced 300% modulus and hardness compared to CH450. These results align with reports on unmodified bio-oils reducing crosslink density by competing with vulcanization agents [5]. The poor dispersion of ESBO further diminished tensile performance. e-SBO and r-SBO mirrored the unaged performance of virgin SBO but showed post-aging declines in SR matrices while remaining stable in NSR systems. In the field of durability and aging resistance, the abrasion resistance of SBO matched that of CH450, while ESBO enhanced ozone resistance due to epoxide functionality but failed to improve wear resistance, likely due to dispersion issues. Virgin SBO and ESBO also enhanced thermal aging stability at full replacement [18]. However, e-SBO and r-SBO showed significant degradation post-aging in SR systems, suggesting matrix-dependent tolerance to degradation products. For dynamic properties, while the loss factor was not directly measured, our green tire prototypes exhibited outstanding wear resistance, rubber cohesion, and oxidative stability in 5000 km road tests. These empirical observations align with Zhu et al. [4] and Xu et al. [5], who reported that improved chain mobility and filler dispersion from bio-oils reduce rolling resistance and enhance grip.

## 4. Conclusions

This study confirms that soybean-derived oils, particularly virgin SBO, are viable replacements for petroleum-based plasticizers in SBR formulations, achieving comparable or superior performance in processing, mechanical strength, and environmental durability. ESBO further contributes to enhanced ozone and tear resistance due to its epoxide structure, while e-SBO and r-SBO offer cost-effective alternatives with acceptable property retention. The successful fabrication and road testing of tires using 100% SBO, exhibiting a burst strength above 1000 kPa and stable performance after 5000 km, demonstrate the practical feasibility of this green material strategy. Moreover, the positive reception and industrial interest generated at TAIPEI CYCLE 2025 underscore the market readiness and commercial potential of this sustainable tire technology. These findings collectively support the advancement of soybean oil-based rubber composites toward industrial-scale adoption and circular economy goals.

Given the current limitations in research resources, including time, instrumentation, and funding, the present study strategically focused on developing environmentally friendly bicycle tires through the full substitution of petroleum-based processing oils with SBO. Building upon prior research, this study systematically evaluated the impact of these bio-based oils on the processability and mechanical properties of rubber compounds, culminating in their successful application to off-road competition mountain bike tires. Future work will expand the evaluation of rubber performance across a broader spectrum of service conditions and characterization techniques. Specifically, deformation strength will be assessed at −50, −30, −10, 10, and 50 °C to better understand temperature-dependent mechanical behavior. In addition, dynamic mechanical analysis will be conducted to quantify the loss factor at 0 °C and 60 °C, representing wet grip and rolling resistance, respectively. To address scientific novelty and deepen material understanding, future investigations will include tribological testing under simulated riding conditions, with a focus on friction and wear resistance. Moreover, the glass transition temperatures (T_g_) of both bio-based and conventional rubber compounds will be determined, offering insight into the thermal and molecular mobility characteristics of these materials.

Finally, iterative formulation refinement will be guided by real-world feedback from competitive cyclists, covering attributes such as traction, durability, handling stability, and riding comfort. Collectively, these efforts aim to bridge application-driven product development with fundamental material science, thereby advancing the sustainable development of high-performance, bio-based green tire technologies.

## Figures and Tables

**Table 1:** Symbols of (non-)staining rubbers with different weight percentages of soybean-derived alternatives.

Weight Percentages of Soybean-Derived Alternatives	Staining Rubber	Non-Staining Rubber
0% SBO + 100% CH450	0-S-SR	0-S-NSR
25% SBO + 75% CH450	25-S-SR	25-S-NSR
50% SBO + 50% CH450	50-S-SR	50-S-NSR
75% SBO + 25% CH450	75-S-SR	75-S-NSR
100% SBO	100-S-SR	100-S-NSR
25% ESBO + 75% CH450	25-E-SR	25-E-NSR
50% ESBO + 50% CH450	50-E-SR	50-E-NSR
75% ESBO + 25% CH450	75-E-SR	75-E-NSR
100% ESBO	100-E-SR	100-E-NSR
100% expired SBO	100-e-SR	100-e-NSR
100% recycled SBO	100-r-SR	100-r-NSR

**Table 2:** A comparison of the properties of the petroleum-based processing oils (CH450) with soybean-derived alternatives.

Oil Property	Specific Gravity	Initial Boiling Point (°C)	Dynamic Viscosity (mm^2^/s, 40 °C)	Absolute Viscosity (cP, 40 °C)	Freezing Point (°F)	Flash Point (°C)	Aniline Point (°C)
CH450	0.928	157	58.0	-	−31.0	198	76.4
SBO	0.941	310.2	59.0	55.5	−31.8	>250	78.9
e-SBO	0.933	314.8	59.6	55.6	−30.7	>250	79.8
r-SBO	0.951	317.8	58.0	55.2	−38.4	>250	63.5
ESBO	0.979	267.2	212.9	208.4	−36.7	>250	47.7

**Table 3:** Rheological tests of the SRs with different weight percentages of soybean-derived alternatives.

Item	Standard	0-S-SR	25-S-SR	50-S-SR	75-S-SR	100-S-SR	25-E-SR	50-E-SR	75-E-SR	100-E-SR
MV value	56–66	54.98	57.78	54.18	57.53	57.7	66.52	60.84	60.16	61.49
TS1 (s)	65–115	68	68	70	70	70	72	77	75	81
TC90 (s)	255–275	241	251	236	249	245	258	265	264	276

**Table 4:** Physical performance of SRs with different weight percentages of soybean-derived alternatives.

Item	Standard	0-S-SR	25-S-SR	50-S-SR	75-S-SR	100-S-SR	25-E-SR	50-E-SR	75-E-SR	100-E-SR
Hardness (HS)	58–64	61	62	61	62	61	62	63	62	61
Tensile strength (MPa)	16–20	18.43	18.39	19.40	18.57	18.10	17.82	17.95	17.34	17.31
M300 (MPa)	5.0–8.0	6.59	6.89	6.87	6.57	6.42	6.59	6.87	6.23	5.60
Elongation (%)	550–750	656.29	672.68	692.95	704.51	707.69	667.33	662.36	675.47	710.60
Tear strength (%)	40–80	70.51	81.51	70.44	75.74	92.76	79.16	72.47	81.75	86.60
Specific gravity	1.18–1.22	1.21	1.22	1.22	1.22	1.22	1.23	1.22	1.22	1.23

**Table 5:** Ozone resistance test and AKRON abrasion loss test of SRs with different weight percentages of soybean-derived alternatives.

Staining Rubbers	Ozone Environment (12 h)	Wear Losses (mL)
0-S-SR	C4	0.21
25-S-SR	C4	0.23
50-S-SR	C4	0.22
75-S-SR	C4	0.20
100-S-SR	eC4	0.22
25-E-SR	C5	0.35
50-E-SR	C5	0.28
75-E-SR	C5	0.27
100-E-SR	B5	0.26

Note: An evaluation standard for the ozone resistance test.

**Table 6:** Decreasing percentage (%) of SRs with different weight percentages of soybean-derived alternatives before and after exposure to ozone environment for 12 h.

Item	0-S-SR	25-S-SR	50-S-SR	75-S-SR	100-S-SR	25-E-SR	50-E-SR	75-E-SR	100-E-SR
Tensile strength (MPa)	4.88	8.17	9.60	11.72	5.80	10.61	12.14	9.48	4.06
M300 (MPa)	−100.96	−112.09	−91.70	−81.34	−87.29	−97.88	−73.65	−77.91	−90.60
Elongation (%)	37.93	46.93	40.62	39.36	37.30	43.57	38.38	36.22	32.76
Tear strength (%)	−6.59	11.69	−4.71	−0.98	18.60	6.14	−1.19	12.74	19.50

**Table 7:** Rheological tests of the NSRs with different weight percentages of soybean-derived alternatives.

Item	Standard	0-S-NSR	25-S-NSR	50-S-NSR	75-S-NSR	100-S-NSR	25-E-NSR	50-E-NSR	75-E-NSR	100-E-NSR
MV value	53–63	67.17	69.92	66.26	68.74	67.59	64.69	67.54	68.48	65.51
TS1 (s)	65–115	89	90	92	91	94	94	94	90	90
TC90 (s)	170–220	207	209	204	204	200	206	206	197	191

**Table 8:** Physical performance of NSRs with different weight percentages of soybean-derived alternatives.

Item	Standard	0-S-NSR	25-S-NSR	50-S-NSR	75-S-NSR	100-S-NSR	25-E-NSR	50-E-NSR	75-E-NSR	100-E-NSR
Hardness (HS)	59–65	66	66	66	66	66	66	65	65	64
Tensile strength (MPa)	11.5–16.5	17.55	17.49	17.01	17.75	17.33	16.88	16.60	17.23	16.50
M300 (MPa)	7–11	9.81	10.46	9.50	9.99	9.28	10.19	9.59	9.77	8.34
Elongation (%)	400–600	476.85	457.51	477.65	480.99	492.85	450.00	465.39	469.78	505.76
Tear strength (%)	30–70	67.04	65.78	64.17	64.08	65.04	68.62	63.45	61.68	56.57
Specific gravity	1.08–1.12	1.114	1.11	1.11	1.12	1.12	1.11	1.12	1.12	1.22

**Table 9:** Ozone resistance test and AKRON wear test of NSRs with different weight percentages of soybean-derived alternatives.

Non-Staining Rubbers	Ozone Environment (12 h)	Wear (mL)
0-S-NSR	N	0.68
25-S-NSR	N	0.66
50-S-NSR	N	0.63
75-S-NSR	N	0.59
100-S-NSR	N	0.56
25-E-NSR	N	0.70
50-E-NSR	N	0.73
75-E-NSR	N	0.77
100-E-NSR	N	0.78

**Table 10:** Decreasing percentage (%) of NSRs with different weight percentages of soybean-derived alternatives before and after exposure to ozone environment for 12 h.

Item	0-S-NSR	25-S-NSR	50-S-NSR	75-S-NSR	100-S-NSR	25-E-NSR	50-E-NSR	75-E-NSR	100-E-NSR
Tensile strength (MPa)	47.34	43.58	49.44	56.45	51.53	53.25	60.59	60.59	52.98
M300 (MPa)	100.00	100.00	100.00	100.00	100.00	100.00	100.00	100.00	100.00
Elongation (%)	53.82	47.60	54.51	61.31	52.20	52.68	61.89	58.88	58.76
Tear strength (%)	33.92	39.36	42.31	46.15	48.55	48.44	55.25	54.19	49.55

**Table 11:** Rheological tests of the SRs and NSRs with e-SBO and r-SBO.

Item	100-e-SR	100-r-SR	100-e-NSR	100-r-NSR
MV value	38.18	37.41	62.92	62.35
TS1 (s)	89	77	94	91
TC90 (s)	233	252	229	204

**Table 12:** Physical performance of SRs and NSRs with e-SBO and r-SBO.

Item	100-e-SR	100-r-SR	100-e-NSR	100-r-NSR
Hardness (HS)	62	64	62	64
Tensile strength (MPa)	18.41	18.90	18.41	18.90
M300 (MPa)	5.15	6.78	5.15	6.78
Elongation (%)	721.29	689.17	721.29	689.17
Tear strength (%)	63.84	71.75	63.84	71.75
Specific gravity	1.15	1.20	1.15	1.20

**Table 13:** Ozone resistance test and AKRON wear test of SRs and NSRs with e-SBO and r-SBO.

Staining Rubbers	Ozone Environment (12 h)	Wear (mL)
100-e-SR	C4	0.23
100-r-SR	C4	0.22
100-e-NSR	N	0.58
100-r-NSR	N	0.54

**Table 14:** Decreasing percentage (%) of SRs and NSRs with e-SBO and r-SBO before and after exposure to ozone environment for 12 h.

Item	100-e-SR	100-r-SR	100-e-NSR	100-r-NSR
Tensile strength (MPa)	35.14	14.66	39.15	36.82
M300 (MPa)	−32.43	−59.29	100.00	60.30
Elongation (%)	34.95	34.85	47.84	41.37
Tear strength (%)	28.74	8.08	31.81	26.82

**Table 15:** BC road test for produced tire (700 × 45C) using 100-S-SR.

Item	Standard	Initial	After Road Test for 5000 km	Difference
Weight (g)	709 ± 21	713	708	−23
Inflation pressure (psi)	30–60	60	50	−10
Outer diameter (mm)	719 ± 6	719.2	718.4	−0.8
Tire width (mm)	45 ± 2.5	46.2	46.6	0.4
Tire section width (mm)	44 ± 2.5	44.9	45.1	0.2
Tire tread arc length (mm)	-	54	55	1

## Data Availability

The original contributions presented in this study are included in the article. Further inquiries can be directed to the corresponding author.

## References

[1] Scott A. (2023). Can tires turn green?. C&EN.

[2] Bijina V., Jandas P.J., Joseph S., Gopu J., Abhitha K., John H. (2022). Recent trends in industrial and academic developments of green tyre technology. Polym. Bull..

[3] Meier M.A., Metzger J.O., Schubert U.S. (2007). Plant oil renewable resources as green alternatives in polymer science. Chem. Soc. Rev..

[4] Zhu X., Peng S.L., Liu Y., Li S., Liu J., Hua J. (2025). Sustainable green tire tread design: Soybean oil-based coupling agent enhances rubber-silica interfacial interaction. Ind. Crops Prod..

[5] Xu H., Fan T., Ye N., Wu W., Huang D., Wang D., Wang Z., Zhang L. (2020). Plasticization Effect of Bio-Based Plasticizers from Soybean Oil for Tire Tread Rubber. Polymers.

[6] Roy K., Poompiew N., Pongwisuthiruchte A., Potiyaraj P. (2021). Application of Different Vegetable Oils as Processing Aids in Industrial Rubber Composites: A Sustainable Approach. ACS Omega.

[7] Moresco S., Scarton C.T., Giovanela M., Carli L.N., Bieliński D.M., Crespo J.S. (2019). Natural rubber compositions with the partial/total replacement of carbon black/naphthenic oil by renewable additives: Rice husk ash and cashew nut oil. J. Appl. Polym. Sci..

[8] Cataldo F., Ursini O., Angelini G. (2013). Biodiesel as a Plasticizer of a SBR-Based Tire Tread Formulation. ISRN Polym. Sci..

[9] Sheldon R.A. (2024). Waste Valorization in a Sustainable Bio-Based Economy: The Road to Carbon Neutrality. Chem. Eur. J..

[10] Kazemi M., Wang H., Fini E. (2022). Bio-based and nature inspired solutions: A step toward carbon-neutral economy. J. Road Eng..

[11] Loizides M.I., Loizidou X.I., Orthodoxou D.L., Petsa D. (2019). Circular bioeconomy in action: Collection and recycling of domestic used cooking oil through a social, reverse logistics system. Recycling.

[12] Oliver-Cuenca V., Salaris V., Muñoz-Gimena P.F., Agüero Á., Peltzer M.A., Montero V.A., Arrieta M.P., Sempere-Torregrosa J., Pavon C., Samper M.D. (2024). Bio-Based and Biodegradable Polymeric Materials for a Circular Economy. Polymers.

[13] Rostami-Tapeh-Esmaeil E. (2023). The Effect of Formulation and Processing Conditions on the Morphology, Physical, Mechanical, and Thermal Properties of Polyolefin Elastomer and Natural Rubber Foams. Ph.D. Thesis.

[14] Prasannakumar P., Sankarannair S., Prasad G., Hari Krishna P.H., Pranav S., Vivek P., Sidharth S., Shanmugam R. (2025). Bio-based additives in lubricants: Addressing challenges and leveraging for improved performance toward sustainable lubrication. Biomass Convers. Bioref..

[15] Dadkhah M., Messori M. (2024). A comprehensive overview of conventional and bio-based fillers for rubber formulations sustainability. Mater. Today Sustain..

[16] Mohamed N.R., Othman N., Shuib R.K., Hayeemasae N. (2023). Perspective on opportunities of bio-based processing oil to rubber industry: A short review. Iran. Polym. J..

[17] Espósito L.H., Marzocca A.J. (2020). Silica-filled S-SBR with epoxidized soybean oil: Influence of the mixing process on rheological and mechanical properties of the compound. J. Appl. Polym. Sci..

[18] Sahakaro K., Beraheng A. (2011). Epoxidized natural oils as the alternative safe process oils in rubber compounds. Rubber Chem. Technol..

[19] Das S., Satpathi H., Roopa S., Gupta S.D. (2021). Sustainability of the tire industry: Through a material approach. Applied Biopolymer Technology and Bioplastics.

[20] Schöbinger M. (2021). Investigations on the Influence of Surface Structure and Modification on Ozone Resistance of Elastomers. Bachelor’s Thesis.

